# A contemporary examination of the effect of driver training for reducing crash risk in novice adolescent drivers: protocol for the DRIVER study, a random assignment trial

**DOI:** 10.1186/s40621-025-00645-2

**Published:** 2025-12-01

**Authors:** Elizabeth A. Walshe, Daniel Romer, Michael R. Elliott, Keith S. Baxelbaum, M. Kit Delgado, Jeffrey P. Ebert, Flaura K. Winston

**Affiliations:** 1 Center for Injury Research and Prevention, Children’s Hospital of Philadelphia, 734 Schuylkill Ave, Floor 13, Philadelphia, PA 19146 USA; 2 Perelman School of Medicine, University of Pennsylvania, Philadelphia, PA USA; 3 Annenberg Public Policy Center, University of Pennsylvania, Philadelphia, PA USA; 4 University of Michigan School of Public Health, Ann Arbor, MI USA; 5 University of Michigan Institute for Social Research, Ann Arbor, MI USA; 6 Department of Biomedical and Health Informatics, Data Science and Biostatistics Unit, Children’s Hospital of Philadelphia, Philadelphia, PA USA

**Keywords:** Injury prevention, Teen Drivers, Motor Vehicle Crashes, Driver Training, Adolescent Development

## Abstract

**Background:**

Motor vehicle crashes and resultant fatalities remain disproportionately high among young drivers, with crash risk peaking immediately after licensure. Although graduated driver licensing laws (GDL) for young novice drivers have reduced such fatalities, driver error remains a leading cause; thus, prevention efforts that target improving skills in novice teen drivers *before* licensure are a strong candidate for reducing crash risk early in licensure. States with more comprehensive driver licensing laws that include mandated driver training before licensure in addition to GDL show lower crash rates post-licensure, but these effects were not determined through rigorous controlled studies of driver training. This paper describes the DRIVER study, a phase III randomized trial that tests the effectiveness of two different driver training programs in reducing young driver crash risk early in licensure in Pennsylvania, a state like many others that does not require formal training for young drivers.

**Methods:**

Learner drivers age 16 and 17 years will be recruited and followed through the GDL learner phase and for six months post-licensure. Participants will be randomly assigned to one of three interventions: professional behind-the-wheel training (n = 333), online hazard training (n = 333), or an active control online vehicle and driver safety course, unrelated to hazard skills training (n = 333). The primary outcomes are on-road crash risk post-licensure (via kinematic hard braking events tracked through a smartphone-based app) and state license examination performance. Secondary outcomes include change in simulated driving performance from baseline to the time of license examination, self-reported and kinematically tracked risky driving behavior (e.g. cell phone use, speeding) and self-reported crashes. Participants will complete baseline surveys and cognitive assessments to determine potential moderating effects of cognitive maturation and risk-taking tendencies.

**Discussion:**

Findings from the DRIVER study will provide insights into training effectiveness generally, and an evidence base for recommendations to policy makers, while also revealing for whom these interventions are less effective.

**Trial registration:**

The study was registered on ClinicalTrials.gov Registry (NCT06413927) in May 2024, https://clinicaltrials.gov/study/NCT06413927 and last updated on August 11th, 2025. This protocol was developed per the SPIRIT (Standard Protocol Items: Recommendations for Interventional Trials) Checklist.

## Key Message

What is already known: States with more comprehensive driver licensing laws that include mandated behind-the-wheel (BTW) driver training before licensure in addition to GDL show lower crash rates post-licensure, but these effects were not determined through rigorous controlled studies of driver training and there may be barriers to accessing BTW training.

What this study adds: This Phase III randomized trial will test the effectiveness of BTW driver training and an evidence-based online virtual hazard skills training in reducing young driver crash risk early in licensure.

How this study might affect research, practice or policy: Findings will provide insights into training effectiveness overall, and an evidence base for recommendations to policy makers, while also advancing research by identifying individual characteristics that determine for whom each type of training is not effective, addressing key gaps in understanding individual differences in training outcomes.

## Introduction

Motor vehicle crashes remain a leading cause of death and injury worldwide, with children and young people most at risk [1]. In the US, young drivers are overrepresented in crashes [2, 3], with long-term sequela including chronic disabilities leading to ongoing medical care, reduced workforce participation, and disability-related costs [4]. The US adopted graduated driver licensing laws (GDL) as the national strategy for reducing young driver crash risk. At a minimum, US GDL requires a learner permit period and restricted driving conditions during an intermediate or junior license [5, 6]. Yet, despite GDL, young driver crashes remain high [7]. Typically, crash risk peaks within the first 2–3 months after licensure, and then lowers to adult levels between 1.5–2 years post-licensure [8, 9]. Furthermore, driver error, due to a lack of skills and experience [10], accounts for approximately 76–94% of crashes [11, 12]. Thus, prevention efforts that target improving skills *before* licensure are promising strategies for reducing crash risk early in licensure.

Recent studies suggest that behind-the-wheel driver training (BTW) may improve safety early in licensure [9, 13]. In Ohio, a state that mandates driver education and BTW in addition to GDL for drivers under age 18 years, these young drivers had better skills at licensure and lower crash rates early in licensure, relative to those licensed at age 18 years (without BTW) [13]. Similar policies and lower crash rates have been observed in California [9]. While these studies were not randomized trial studies, and could not account for exposure (i.e. varying time spent on the road), they show some promise that skills training before licensure may be a modifiable risk factor. However, a 2022 review of state policies found that 17 states (including Pennsylvania) have no BTW requirements for young drivers, with an additional 3 states allowing for BTW to be replaced (e.g. with adult supervised practice) [14].

State endorsement of BTW for licensure has dwindled since the 1980s, since the only random assignment study of BTW to date (the DeKalb study, 1983) [15] found modest short-term protective effects of BTW on 6 month outcomes, but concluded it did not reduce crashes overall [15, 16]. The most rigorous re-analysis of these data showed a 13.1% crash reduction in the first 6 months of licensure (which we now know is the period of highest lifetime crash risk [8, 17]). Prior research shows that skill deficits underscore most of these early crashes, pointing to the potential value of training [10, 11, 18–20]. While the DeKalb study was rigorous in its random assignment design, it did not capture exposure, and predates modern GDL, vehicle safety, driver training standards, as well as modern risks (e.g. cell phone use). Therefore, there is a critical need for a contemporary examination of the effectiveness of driver training for improving young driver safety in the US.

The DeKalb study also did not account for other known individual risk factors, such as cognitive development and impulsive traits. Even among typically developing youth, executive function and attentional skills, and associated brain regions, show dramatic maturation during adolescence (when many adolescents learn to drive) before stabilizing by age 18–19 or later [21]. These executive functions, particularly working memory, have been linked to driving performance and outcomes in young drivers [22–25]. Risk-related impulsive personality traits that peak in adolescence in association with executive function have also been associated with crash risk [26–29]. However, we hypothesize that sufficient skills training may offset developmental risk factors, given the youngest new drivers in Ohio who completed driver training had the lowest crash rates [13].

Despite the potential benefits of BTW, access is uneven due to cost and location barriers [14, 30]. Alternatively, validated digital training programs for improving safety–critical skills and reducing crashes have the potential to overcome these barriers. Specifically, three free virtual training programs in hazard awareness and mitigation (RAPT and ACT) [31–33], and attention maintenance skill training (FOCAL) [34] have been packaged as an omnibus driver education program (ACCEL) [35] that has shown promise in licensed young novice drivers [36]. Online driver's education programs can eliminate the need for travel to a physical driving school, benefiting individuals in remote or rural areas where access to such facilities is limited. However, this intervention has not been tested in the pre-licensure phase for efficacy in reducing crash risk post-licensure.

### Objectives

The primary objective of the DRIVER (DRIVer Education Research) study is to quantify the extent to which modern online driver training and BTW prior to licensure can add protection beyond GDL against crash risk early in licensure. The secondary objective is to quantify the effect of driver training on skill acquisition. The study aims and outcomes are summarized in Table 1. We hypothesize that both ACCEL and BTW training will reduce crash risk and improve driving skills compared to the control group. More specifically, we hypothesize that ACCEL will show greater improvement in crash risk and skills than BTW on the theory that ACCEL is more targeted to the critical reasons for crashes among novice adolescent drivers. However, we expect BTW to perform better on PA license examination outcomes since this test measures skills directly trained by BTW. We also hypothesize that those with weakness in cognitive and attentional control will benefit most from the training interventions in the key outcomes, and those with personality differences and risky driving practices linked to crash risk may be more resistant to the effects of driver training.

**Table 1:** Summary of study aims and associated outcomes

Study Aim	Primary Outcome	Secondary Outcome
**Aim 1**. Determine the effect of driver training (ACCEL or BTW) compared to GDL alone on post-licensure crash risk	Hard Braking Events per 100 miles driven via Way to Drive monitoring app	• Phone Use while Driving (minutes per hour of driving) • Speeding (proportion of time > 10 miles over posted speed limit) • Self-reported crash involvement • Self-reported risky driving behavior
**Aim 2**. Determine the effect of driver training (ACCEL or BTW) compared to GDL alone on skill acquisition	Pass rate on the first attempt at the state licensing examination	• Virtual Driving Assessment (VDA) Performance at T2 • Change in T1-T2 VDA performance • Number of license exam attempts
**Aim 3**. Identify age-related risk factors for crashes that may moderate the response to training	Interactions with Aim 1 & 2 outcomes	Interactions with Aim 1 & 2 outcomes

### Trial design

**Fig. 1:**
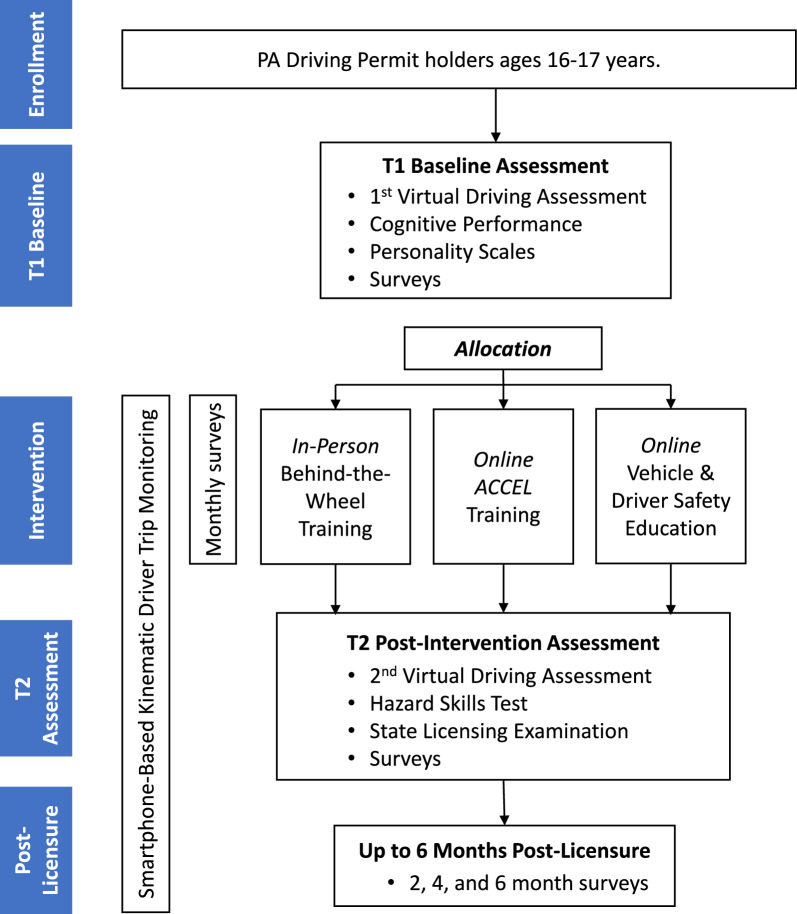
Flow diagram of participant progression through the trial, with participation estimated between 8–18 months (with varying learner/intervention phase)

This trial uses random assignment with three parallel groups and follows an individually randomized group-treatment (IRGT) design. Participants are enrolled early in their learner permit and followed through licensure, and up to 6 months post-licensure, with the primary end point of crash risk at 2 months post-licensure. Participation length will vary with permit duration (range 8 to 18 months). After baseline, participants are randomized 1:1:1 to one of three groups (n = 333 per group): (i) professional BTW; (ii) ACCEL, a modern online driving skills training program; (iii) an active control online program covering vehicle safety topics without skills training. Participants in the BTW group will be clustered by instructor to account for variation in driver teaching style. Participants will be monitored remotely through the learner and post-licensure phases using surveys and a smartphone application capturing driving kinematics to quantify exposure and crash risk. At licensure, driving skill will be measured using three methods: the virtual driving assessment (VDA), an online hazard skills test, and results from the license examination. The primary endpoint is on-road kinematic crash risk (the rate of hard braking events) at 2 months post-licensure, with continued follow-up through 6 months post-licensure. Kinematic risky driving (KRD) events such as hard-braking events, have previously been validated as behavioral proxies for elevated crash risk [37–42]. See Fig. 1 and Table 2 for participant flow through the study and all procedures and measures through the trial.

**Table 2:** Outline of the schedule of enrollment, interventions, and assessments

Timepoint	Enrollment	Baseline	Allocation	Intervention						Post-intervention	Post-Licensure		
	*-t*_*1*_	*t*_*1*_	*0*	*m*_*1*_	*m*_*2*_	*m*_*3*_	*m*_*4*_	*m*_*5*_	*m*_*6*_	*t*_*2*_	*m*_*8*_	*m*_*10*_	*m*_*12*_
Enrollment:	*X*												
Eligibility Screen	X												
Informed Consent/Assent	X												
Allocation to Trial Arm			X										
Interventions:													
Active Control Online Education				X	X	X	X	X	X				
ACCEL Online Driver Skills Training				X	X	X	X	X	X				
Behind-the-Wheel Training				X	X	X	X	X	X				
Assessments:													
Virtual Driving Assessment		X								X			
Penn Neurocognitive Battery		X											
Day-of-Performance Factors		X											
Driving Intake (status)		X											
Driving History (crashes/citations)		X		X	X	X	X	X	X	X	X	X	X
Personality Scales		X											
Demographic & Household Factors		X								X			
Adolescent Health Risk Factors		X											
Way to Drive Monitoring													
Driving Practice Quality Questionnaire				X	X	X	X	X	X	X			
Hazard Skills Test										X			
State Licensing Examination										X			
Driver Behavior Surveys										X	X	X	X

This table outlines the schedule of study procedures and provides an approximate length of participation, however, the intervention phase length will vary, and some drivers may need to retake the state license examination, extending the time before post-licensure follow-up. T1 and T2 = study assessment timepoints before and after the intervention learner permit phase; m = month

## Methods & analysis

This study is registered at ClinicalTrials.gov as of May 2024 (NCT06413927). Enrollment started in August 2024 and data collection is ongoing with an expected primary completion in December 2027, or until all final data collection is complete.

### Patients and population

The trial aims to enroll up to 1200 adolescent learner drivers with a goal of achieving 1000 evaluable participants, accounting for 20% attrition. Participants will primarily be recruited from primary care clinics in the Children’s Hospital of Philadelphia (CHOP) Network in the southeastern region of Pennsylvania (PA). The CHOP Network serves a heterogenous patient population: among patients age 16–17 years that visited a clinic at least once in the past 2 years, 33.7% are insured via Medicaid and the Children’s Health Insurance Program (CHIP); 49.4% are female sex; and approximately 47.9% are non-white. Potentially eligible adolescents will be identified through the electronic health record (under a waiver of HIPAA authorization for screening). Other eligible learner drivers who attend other Primary Care clinics and/or who respond to other recruitment and advertising strategies (e.g., study fliers) and who agree to travel to study locations for in-person procedures may also be enrolled.

#### Eligibility criteria

The eligibility criteria for this study include the following: participants must be aged 16–17 years, reside in PA, hold an active PA Learner’s permit with less than 30 h of driving practice (state requires 65 h before licensure), and have never taken a state driving licensing examination, and plan to get a license and to have access to a vehicle to drive after licensure. In addition, participants must have a personal cellphone, access to a smartphone/computer with internet access, and the ability to read and write in English to complete study tasks. Of note, prior research indicates that 95% of adolescents possess smartphones, with no significant differences by demographics [43]. Non-PA residents, non-English speaking participants, and those already enrolled in other learner driver studies will be ineligible.

### Intervention descriptions

All trial participants will receive care as usual, including information to access a Parents Supervised Driving Program from the Pennsylvania Department of Transportation (PennDoT), as well as information on how to access the PennDoT recommended smartphone app for logging practice hours (RoadReady App). In addition, one group will receive professional BTW with a local driving school instructor (6 h: 4 × 1.5 h lessons), another group will receive ACCEL online hazard skills training (~ 2 h), and another will receive an online active control vehicle and driver safety program (~ 2 h, with no skill training). While the ACCEL intervention is shorter than BTW instruction, this program is designed to be highly interactive and cognitively engaging, focusing specifically on the skill deficits that cause young driver crashes, and thus potentially offsetting the shorter duration. While the online programs can be completed in one sitting, participants can also take breaks and resume where they left off. Adherence (progress and duration) will be tracked via the e-learning platform (ScormCloud) event log and via a quiz and test at the end of the training. For BTW, driving instructors will fill out a brief post-lesson survey to confirm lesson content covered and completion.

#### In-person behind-the-wheel driver training (BTW)

Professional BTW will be carried out by a third-party accredited driver training school, and their partners. The driving school was chosen for its dual certification for training and license testing and is audited every 6–12 months, giving further support for the integrity of the intervention. The standardized curriculum used for the 6 h of training meets Pennsylvania and national standards, as reviewed by subject matter experts external to the study team from the PA Department of Education, Ohio Traffic Safety Office, and representatives of the American Driver and Traffic Safety Education Association (ADTSEA). Certified PA driving instructors will deliver the training, and school leadership will oversee fidelity through supervision, observations and end-of-lesson surveys completed in REDCap. Staff will also complete brief REDCap surveys (onboarding, post-lesson, offboarding) to monitor student progression and curriculum adherence.

#### Online driver skills training (ACCEL)

ACCEL [35] is an evidence-based online training program developed by Donald Fisher and colleagues at the University of Massachusetts Amherst. Designed to improve hazard anticipation, recognition, and attention maintenance, prior studies have shown ACCEL improves driving performance and reduces crash risk [31–33, 36]. For this trial, ACCEL was adapted for remote delivery to teen learner drivers by CHOP’s Department of Global Pediatric Education, with input from the CHOP Parent Family Education office to enhance health literacy. Pilot testing with local high school students informed revisions to language and graphics. The final version was reviewed and approved by the original developer, Donald Fisher, and takes about 2 h to complete. In order to track remote adherence, progress and performance, this interactive training is hosted on SCORMCloud (by Rustici Software LLC), an online e-learning platform that can be accessed from any computer with internet access.

#### Active control: online vehicle and driver safety education

The active control group will complete an online video-based education program (~ 2 h) that covering vehicle maintenance and occupant safety (i.e., car seats, seat belts) but no driving skills training content. A short quiz at the end will confirm compliance. This program is also hosted on SCORMCloud which automatically tracks adherence, progress, and completion.

### Variables and outcome measures

Table 1 provides a summary of the study aims and outcomes. Table 2 summarizes all study procedures and measures.

#### Primary outcomes

There are two primary outcomes. First, crash risk in the first 2 months of licensure (Aim 1 and 3) will be measured as the rate of hard braking events per 100 miles driving, captured via the Way to Drive smartphone app. Monitoring of hard braking events will continue through 6 months to assess crash risk at 6 months also. The app, provided by Cambridge Mobile Telematics, deploys the same algorithms as leading usage-based automobile insurance programs to measure risky driving behaviors (phone use during trips, speeding, and other kinematic risky driving measures) [38]. Second, driver skills at licensure (Aim 2 and 3) will be measured as the pass rate on the first attempt of the state licensure examination. While the state licensing exam assesses a limited set of procedural driving skills, it provides a standardized, real-world benchmark of basic driving competence and is a relevant proximal outcome for novice drivers.

#### Secondary outcomes

Secondary outcomes include measures of risky driving behavior post-licensure. Self-reported crash involvement captured in follow-up surveys at 2-, 4- and 6-months post-licensure will provide a secondary outcome of crash risk. Self-reported risky driving behavior will also be captured in follow-up surveys. Other indicators of risky driving behavior post-licensure captured from the Way to Drive app include phone use while driving (number of minutes per hour driving) and speeding (proportion of driving at speed > 10 miles over the speed limit). Secondary outcomes of driver skills at the time of licensure will be measured via driver performance on the state licensing examination and change in performance class (pre- vs post-intervention) on the validated VDA [44, 45]. The number of license examination attempts and skills on the on-road examination will also be recorded as a secondary outcome.

#### Other measures

At baseline, participants complete: (i) a validated VDA [44, 45]; (ii) executive function tasks of attention and working memory via the validated University of Pennsylvania Computerized Neurocognitive battery (Penn CNB [21, 46]: specifically the Penn Continuous Performance Test, the Letter N-Back test, Penn Go/No-Go task, and the Penn Abstraction and Working Memory task); (iii) a day-of performance factors survey (sleep/restfulness, caffeine and medication intake, and mood via the Positive and Negative Affect Schedule scale [47]); (iv) two impulsive personality scales: *acting-without-thinking* from the Eysenck Personality Inventory, Impulsivity Subscale [48]; and Temporal Discounting or Delay Discounting via a standard monetary reward task for children [49] (v) a Sensation Seeking scale (4-items form of the Zuckerman’s Sensation Seeking Scale [50]); (vi) a survey of demographic and household factors (vehicle access and features, living situation and household financial status indicators); (vii) items from the adolescent health risk questionnaire (risk behaviors, relationship with parents); (vii) a driving history survey (crashes and citations). In addition, demographic data from the electronic health record will be collected: age, sex, child opportunity index (COI) scores as neighborhood measures of financial status and opportunity [51]. 

During the intervention phase (i.e., learner permit to licensure), participants will receive monthly surveys to ask about their learning to drive (via the driving practice quality questionnaire [52]) and crashes and citations. Participants’ driving exposure (a confounder) will also be passively monitored via the Way to Drive app. This also has an algorithm that predicts likely crash events, which will be analyzed retrospectively as an exploratory outcome. Intervention adherence will also be tracked (via driving instructor progress reports and online e-learning platform data for online intervention progress and completion).

At the end of the learning/intervention phase, at the point of licensing examination, all participants will complete the following: (i) a second VDA; (ii) PA state licensing examination; (iii) online hazard skills test; (iv) self-report surveys of driving experience (crashes and citations), driving habits and behavior (modified driver behavior questionnaire (mDBQ) [22, 27, 53]; (vi) aggressive driving scale [54, 55]; Youth Risk Behavior Survey [55] Driving items, driving confidence scale [56]); (vii) driving practice quality questionnaire and any other ways they learned to drive beyond the interventions prescribed; and (viii) updated demographic and household factor information (update since T1). Participants who do not pass the state licensing exam initially will be given the opportunity to retake the exam for free and to continue in the trial. All participants who receive a license and remain for post-licensure phase will continue to have their driving trips monitored by the *Way to Drive* and complete surveys at 2, 4, and 6 months, capturing driving behaviors, confidence, crash history, and vehicle access (as at T2 and noted above). We will use multiple imputation and sensitivity analyses to address missing data, and we will report adherence rates for each intervention and for survey completion.

### Sample size

The target sample size for this randomized controlled trial is 1000 evaluable participants, with approximately 333 in each study arm (one group may include 334). This is based on the primary hypothesis of a reduction in hard braking events with driver training. The trial is powered to detect a 20% reduction in hard-braking events, a threshold identified by the U.S. Department of Transportation as predictive of reduced crash risk [57]. Additionally, it is designed to detect differences in state licensure exam pass rates between groups ranging from 15 to 19%, as well as an absolute increase of 10% to 13% in the proportion of drivers assigned to safer skill categories based on VDA classification. These thresholds reflect meaningful improvements in driving safety. A priori power analysis accounted for up to 20% attrition while maintaining 80% power to detect clinically meaningful effects, resulting in a worst-case scenario of 266 participants per arm. Retention strategies and participant replacement procedures are expected to mitigate attrition, leading to a final sample size of n = 800–1,000.

#### Recruitment

Adolescent learner drivers will be recruited through multiple channels. Patients identified by the Electronic Health Record (EHR) that meet the age criteria and who have an upcoming clinical visit will be contacted by the study team in advance or approached on site at the Primary care clinic visit, to share information about the study and eligibility criteria. Additional candidates may be identified through retrospective review of Adolescent Health Questionnaires indicating intent to drive within 12 months (conducted during annual adolescent wellness visits). The study team will also utilize CHOP recruitment services, the *Recruitment Enhancement Core (REC)*, to expand outreach through the EHR (EPIC). Adolescents from other CHOP primary care clinics or who respond to study fliers will also be contacted about the study. Following an initial recruitment call, written parent informed consent and child assent will be obtained, along with HIPAA authorization, via the secure REDCap electronic consent platform. Once enrolled, participants will be scheduled for baseline study visit procedures, before allocation.

### Allocation

After enrollment and baseline assessments, trial participants will be randomized into one of three cohorts in a 1:1:1 ratio so that there will be approximately 333 subjects in each arm, stratified by equal-size age categories (16 and 17) per arm. Randomization will be automatically conducted via the OnCore Clinical Trial Management System (CTMS). Allocation will be made by randomly generating block sizes of 6 and assigning individuals within an age category using the block assignments as they become available for randomization; this will be repeated until all individuals within an age group have been assigned to one of the three intervention arms.

### Blinding

Subjects cannot be blinded to the intervention arm assignment due to the nature of the interventions (e.g., online versus behind the wheel training). For analysis, all primary outcomes are assessed in a way that is blind to intervention assignment. The Way to Drive app provides hard-braking events via automated data coding algorithms. Similarly, VDA performance is determined based on previously validated algorithms to identify driver categories [44, 45]. State license exams are conducted by independent examiners who are blinded to intervention group assignment. This is standard procedure, per state third-party testing regulations. Research staff administer the baseline surveys and other procedures before participants are allocated to an intervention arm. The cognitive battery at baseline will be electronically delivered, with accuracy and reaction time automatically recorded and scored by Penn CNB (external to the study team).

### Data collection and management

The secure REDCap platform will be used for survey delivery, data storage, and collecting participant progress reports from driving school staff, as well as communication logs and study visit notes. CHOP’s CTMS will support enrollment, randomization, and participant tracking. The Department of Bioinformatics and Health Information (DBHI) and Arcus (data management and archiving service) at CHOP will provide expert data management for this trial. They will receive, manage, and link the data sets that will be honest brokered before analysis. This ensures that the research team conducting the main study analyses does not have access to any personally identifying information in the analysis dataset, while still being able to conduct the necessary analyses for the study.

### Statistical methods

Primary and secondary outcomes will be analyzed by intention-to-treat, with each outcome assessed with a separate model. Results will be interpreted based on both statistical significance (α = 0.05) and effect size, emphasizing clinically meaningful differences. As a secondary analysis, baseline covariates will be included in the model, including age at enrollment, sex, and initial VDA class. Additional factors will be examined as potential confounders (e.g., time between first VDA and licensure) and mediators (e.g., number of practice trips, miles driven, and self-reported practice quality).

To address any non-compliance in the intervention, we will use instrumental variable (IV) analysis [58], which treats the randomization assignment as the instrument. We will also consider principal stratification [59], which can relax some of the assumptions about instrumental variable analysis such as no effect of intervention assignment independent of intervention. Both of these methods attempt to estimate intervention effects among young drivers who would comply with intervention assignment regardless of assigned intervention arm. Compliance will be defined as completing more than 2/3 of the training; in the BTW arm, this would entail completing two of the three lessons. In the ACCEL and active control arms, this would entail completion of at least 2/3 of the content. In follow-up analyses, we will include the proportion of training received as a continuous measure, operationalized as the percentage of lessons completed in the assigned intervention, and utilize the mediation analyses, treating training received as a form of mediation. In all analyses we will consider log or power transformations to reduce skewness in the outcome.

Attrition is expected to be modest given the trial's retention strategies, for an estimated final sample size between 800 and 1000 participants. Participants lost before or during the intervention phase may be replaced. Participants who drop out after intervention but before the licensure exam will be encouraged to complete a second VDA. Data for available outcomes to this point will be considered for these participants, and outcomes that can be feasibly imputed will be included in analysis. For participants who fail their initial licensure exam, the study will fund a second attempt and if they pass, they will continue in the trial and follow-up data will be collected. For the rare individuals who fail the second attempt, if they go on to pass on a subsequent attempt (not provided by the study) within the study window, their follow-up data will be collected post-licensure. If the participant does not pass their licensure exam within the study window, they will not be replaced; instead, data for available outcomes will be considered for analyses, with other outcomes imputed as feasible using the data sources noted above.

### Monitoring and oversight

The data and safety monitoring plan for this study will comprise monitoring by the PIs and Co-investigators, IRB oversight, and an independent Safety Monitoring Committee (SMC) for monitoring the trial. The risk for this clinical trial is considered minimal. However, to ensure an added layer of protection for our study and its participants, an external Safety Monitoring Committee (SMC) was appointed to review and monitor the study procedures, patient enrollment, and number and nature of adverse events. All significant adverse events will be reviewed by SMC to determine if additional safety measures should be initiated. No interim analyses of the proposed research aims are planned. Given the nature of the intervention and the primary outcomes being measured post-licensure, interim analyses are not feasible or informative. However, we will conduct ongoing data quality checks and monitoring.

#### Harms

Participants are informed during consent of the potential for simulator sickness while using the VDA, although this event is expected to be rare (previously, < 2% experience this during the VDA). Symptoms are monitored during the virtual driving assessment, and participants may discontinue if discomfort occurs. Given the minimal risk procedures, serious adverse events are not expected. Any unanticipated problems will be reported to the SMC and CHOP IRB. Other adverse events will be tracked and documented.

## Ethics and dissemination

This study was initially reviewed approved by the CHOP IRB on 02/17/2023 (IRB 21–019445). Results will be published in peer-reviewed journals and disseminated at national and international meetings, with possible media press releases. We will follow the Consolidated Standards of Reporting Trials (CONSORT) guidelines for reporting the results of parallel arm trials.

### Changes to protocol

To meet recruitment targets, recruitment strategies were expanded to include email campaigns to eligible patients, social media, and community fliers.

### Patient and public involvement statement

The ACCEL training was refined for health literacy with input from CHOP’s Parent Family Education Office and pilot tested through cognitive interviews with local teens. Their feedback informed language and instructional design improvements.

## Conclusion

This study is the first random assignment trial of driver training conducted in four decades. It compares behind-the-wheel training, online hazard skill training, and an active control online intervention for reducing crash risk early in licensure. The inclusion of real-world outcome measures ensures the findings will have practical relevance for improving driving safety. By integrating cognitive and personality measures, the study aims to identify for whom each type of training is effective and for whom it is not, addressing key gaps in understanding individual differences in training outcomes. In addition, follow-up surveys in the licensed phase provide some measures of self-reported crashes, driving confidence, aggression, and driver behaviour habits. While *locus of control* and crash beliefs were not included in our surveys, we recognize their potential value and will consider incorporating them in future follow-up studies or exploratory analyses. In addition, while the study is limited to English speaking individuals in one region, the results nonetheless have the potential to inform evidence-based policies and contribute to the design of targeted, multifaceted driver training programs that enhance safety and effectiveness.

## Data Availability

No datasets were generated or analysed during the current study.
